# Metataxonomic analyses reveal differences in aquifer bacterial community as a function of creosote contamination and its potential for contaminant remediation

**DOI:** 10.1038/s41598-019-47921-y

**Published:** 2019-08-13

**Authors:** Aline Daniela Lopes Júlio, Ubiana de Cássia Mourão Silva, Julliane Dutra Medeiros, Daniel Kumazawa Morais, Vera Lúcia dos Santos

**Affiliations:** 1 Laboratory of Applied Microbiology, Microbiology Department, Institute of Biological Science, Universidade Federal de Minas Gerais, Belo Horizonte, MG Brazil; 2 Biosystems Informatics and Genomics Group, René Rachou Research Center/Fiocruz-MG, Belo Horizonte, MG Brazil; 3 Laboratory of Environmental Microbiology, Institute of Microbiology of the Czech Academy of Sciences – CAS, Prague, Czech Republic

**Keywords:** Environmental biotechnology, Classification and taxonomy, Applied microbiology, Water microbiology, Environmental impact

## Abstract

Metataxonomic approach was used to describe the bacterial community from a creosote-contaminated aquifer and to access the potential for *in situ* bioremediation of the polycyclic aromatic hydrocarbons (PAHs) by biostimulation. In general, the wells with higher PAH contamination had lower richness and diversity than others, using the Shannon and Simpson indices. By the principal coordinate analysis (PCoA) it was possible to observe the clustering of the bacterial community of most wells in response of the presence of PAH contamination. The significance analysis using edgeR package of the R program showed variation in the abundance of some Operational Taxonomic Units (OTUs) of contaminated wells compared to uncontaminated ones. Taxons enriched in the contaminated wells were correlated positively (p < 0.05) with the hydrocarbons, according to redundancy analysis (RDA). All these enriched taxa have been characterized as PAH degrading agents, such as the genus *Comamonas, Geobacter, Hydrocarboniphaga, Anaerolinea and Desulfomonile*. Additionally, it was possible to predict, with the PICRUSt program, a greater proportion of pathways and genes related to the degradation of PAHs in the wells with higher contamination levels. We conclude that the contaminants promoted the enrichment of several groups of degrading bacteria in the area, which strengthens the feasibility of applying biostimulation as an aquifer remediation strategy.

## Introduction

Over the past few decades, a large number of pollutants have been released into the environment due to the rapid expansion of various human activities, mainly industrial ones^1^. One of these compounds is creosote oil, a thick, amber-to-black oily liquid obtained by fractional distillation of crude coal tar^2,3^. This compound has been used as the main preservative of wood structures such as railway sleepers for more than a century, acting as an impregnation agent^4,5^. Creosote is a complex mixture of more than 200 persistent organic compounds^6,7^. Its composition varies according to the origin of coal and manufacturing processes, but generally is composed of approximately 85% polycyclic aromatic hydrocarbons, 10% phenolic compounds and 5% N, S and O heteroaromatics^2,8^, besides the monoaromatic hydrocarbons benzene, toluene, ethylbenzene and xylenes (BTEX)^9^. This contamination type deserves further attention given that about 20–40% of creosote-prevalent PAHs correspond to the 16 PAHs listed as priority pollutants by the US-EPA (United States Environmental Protection Agency), due to their recalcitrant, bioaccumulative character and their toxic, genotoxic, immunotoxic and carcinogenic properties^2,7,10^.

The widespread use of creosote along with inadequate disposal practices has led to severe worldwide contamination, mainly of surface soils and groundwater^6,9,11^. Once PAHs reach the ground, their partition into the different components of this system depends on their solubility in water and evaporation in the air, so that they can firmly adsorb to soil particles, evaporate or migrate to the groundwater^2,12–14^. Those components that do not dissolve in water are called non-aqueous phase liquids (NAPLs) and these liquids are subdivided in two classes according to density: those more dense than water, dense non-aqueous-phase liquids (DNAPLs) and those less dense than water, light nonaqueous-phase liquids (LNAPLs)^15^. Although NAPLs exist in subsurface as a separate liquid phase, they slowly dissolve in flowing groundwater, giving rise to aqueous-phase plumes that can migrate, spreading these contaminants over a wide area^16^. As a result, contaminated soils and groundwater are often found in places close to recent and old treatment facilities. It should be assumed that if restoration measures are not carried out, these sites will continue to emit pollutants for a long period, representing a reservoir and a continuous source of distribution of these contaminants^17^.

Therefore, creosote residues represent a threat to all biotic compounds of ecosystems and the contaminated sites are considered a priority for remediation^4,6,10^. Among the available remediation technologies, the use of microorganisms with ability to degrade and detoxify environmental contaminants, named bioremediation, has been highlighted^18,19^. Bioremediation is an appealing approach to dealing with environmental contaminants as it often results in removal of a contaminant through natural biological processes. Diverse studies have revealed that, under certain conditions, most PAHs are susceptible to microbial degradation^10,20^, including bacteria belonging to the *Pseudomonas*, *Acinetobacter*, *Sphingomonas*, *Bacillus*, *Enterobacter*, *Mycobacterium*, *Rhodococcus* and *Arthrobacter* genus^10,21^. This fact has led to an increase in the application of the bioremediation approach in the restoration of environments contaminated by PAHs^7,22,23^.

Subsurface environments are some of the largest habitats for prokaryotes, and their total biomass probably exceeds their numbers in the rest of the biosphere^24,25^. However, few studies reported the potential use of *in situ* bioremediation in the decontamination of hydrocarbon-contaminated aquifers, because little is known about hydrocarbon degrading microbiota or the impacts of anthropogenic contamination on the microbial community structure and functional composition^26–28^. In contaminated environments, members of the indigenous microbiota showing tolerance and catabolic potential to use PAHs as a source of carbon and energy are selected, constituting the base for natural attenuation of these sites^7^. This microbiota is the target of the biostimulation technique that consists in the adjustment of organic and inorganic nutrients and physic-chemical parameters to stimulate microbial growth, as well as interventions to increase the bioavailability of the contaminants^29,30^. It can also be prospected regarding as catabolic potential, important for the development of inoculants^7^, which have already been successfully used in hydrocarbon bioremediation field-scale studies^22,31^. At Brazil, there are more than 50 biological commercial products registered by the Brazilian Institute of Environment and Renewable Natural Resources (IBAMA, 2018)^32^ for use in remediation, mainly of water effluents but also of agroindustry solid waste, lagoons, sewage and sites contaminated with petroleum derivatives. These products include patented inoculants or authorized for use as Know how technology.

There are a number of ways to assess the composition and function of microbial communities in aquifer samples. Cultivation and isolation of microorganisms is the traditional method, but less than 10% of the bacteria are cultivable using standard cultivation^33,34^. On the other hand, the application of molecular methods, such as quantitative real-time PCR and metataxonomic analyzes with the sequencing of marker regions, such as 16S rRNA, offers the possibility of detecting uncultivable members of microbial communities, as well as predicting their functions^34^. With the rapid advances in the next generation sequencing technology, its cost has significantly reduced and the scale of sequencing projects has been increased accordingly^19^.

Therefore, these methods provide an opportunity to monitor environmental microbes and greatly expand our knowledge of the microbial processes involved in bioremediation^35^. They also allow us to understand the response of the aquifer microbiota to multiple stressors^36–38^. In addition, predictive tools such as PICRUSt (Phylogenetic Investigation of Communities by Reconstruction of Unobserved States) make it possible to estimate the composition of functional gene families in the metagenome of the environment under study^39^. This tool helps us to infer the potential of the local communities to degrade contaminants and to support *in situ* bioremediation. This information is important in guiding the choice of the physic-chemical conditions needed to accelerate the activity of specific catabolic groups or even the need to strictly plan the addition of microbial inoculants. Thus, the objective of the present study was to compare the bacterial community structure of creosote-contaminated and non-contaminated aquifer liquid fraction samples using metataxonomic analyses and to infer the community’s potential to degrade contaminants *in situ* beyond the feasibility to implement biostimulation.

## Results

### Chemical analysis of aquifer wells

**Figure 1:**
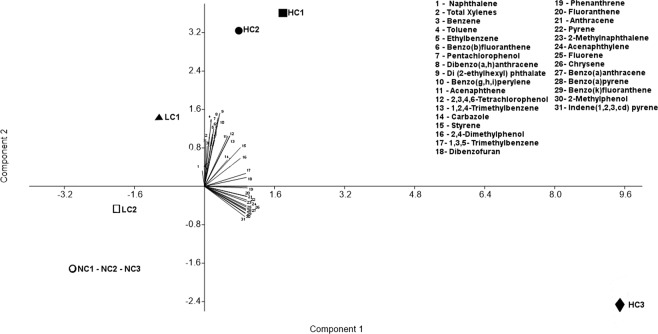
Principal component analysis (PCA) of the aquifer wells VOC and SVOCs concentrations. The axes, corresponding to the contaminants, were numbered in the clockwise direction. The different wells are represented by different symbols except the three non contaminated (NC) wells.

The samples collected in the wells were characterized in relation to the concentration of volatile organic compounds (VOCs) and semi-volatile organic compounds (SVOCs) totaling 31 different contaminants. In three wells, contaminants were not detected, while in the others a wider range of concentrations was found (Table 1). In this way, three groups of wells were defined according to the concentration of these contaminants: three wells without contamination (NC1, NC2 and NC3), three wells with high concentrations of most compounds (HCl, HC2, HC3) and two wells with low concentrations of most compounds (LC1 and LC2). By multivariate principal component analysis (PCA), it was possible to find the VOCs and SVOCs accounting for the maximum variability of the data at well level (Fig. 1). Of the total variability 79.7% was explained by the two principal components (PC1, 60.7% and PC2, 19%). The hydrocarbons that most contributed to PC1 were: Phenanthrene, Anthracene, Fluoranthene, Dibenzofuran, Fluorene, 2-Methylnaphthalene, Pyrene, Acenaphthylene, Benzo(k)Fluoranthene, Benzo(a)Anthracene, Benzo(a)pyrene, Chrysene and 1,3,5-Trimethylbenzene (mostly SVOCs), and the hydrocarbons that most contributed to PC2 were Naphthalene, Total Xylene, Benzene and Toluene (mostly VOCs). We could observe the group formed by wells NC1, NC2 and NC3, which overlap, located in the negative part of the two components. The HC3 well, although also having a high concentration of the contaminants, stayed in a different group of the HC1 and HC2 wells because of its higher concentration of SVOCs and lower concentration of VOCs. Although LC1 and LC2 wells were close together in the PCA, they stayed somewhat separated in component 2 because of their differences in contaminant profile, since the LC1 well had higher concentrations of naphthalene and total xylene, two of the contaminants that most contributed to PC2, as already mentioned.

**Table 1:** Chemical analysis of volatile organic compounds (VOCs) and semi-volatile organic compounds (SVOCs) in the eight aquifer collected samples.

VOC (volatile organic compounds)	HC1	HC2	HC3	LC1	LC2	NC1	NC2	NC3	Research value set by CONAMA* 420 (μg/L)
	(µg/L)	(µg/L)	(µg/L)	(µg/L)	(µg/L)	(µg/L)	(µg/L)	(µg/L)	—
1,2,4-Trimethylbenzene	148	71.9	135	42.2	1.8	—	—	—	15
1,3,5- Trimethylbenzene	82	44.6	126	1.8	1.3	—	—	—	8.7
Benzene	4820	1170	15.1	746	450	—	—	—	5
Styrene	<1.0	52.9	28.6	<1.00	<1.00	—	—	—	20
Ethylbenzene	216	9.6	11.1	358	<1.00	—	—	—	300
Toluene	105	380	40	5.4	3.6	—	—	—	—
Total Xylenes	1256	552	192.1	126.7	110.1	—	—	—	500
**SVOCs (semi-volatile organic compounds)**	**(µg/L)**	**(µg/L)**	**(µg/L)**	**(µg/L)**	**(µg/L)**	**(µg/L)**	**(µg/L)**	**(µg/L)**	—
2,3,4,6-Tetrachlorophenol	<1.0	2.36	1.1	<0.10	<0.10	—	—	—	10.5
2,4-Dimethylphenol	12.6	<1.00	<1.00	169	<1.00	—	—	—	27
2-Methylphenol	<1.0	<1.00	<1.00	<1.00	<1.00	—	—	—	—
2-Methylnaphthalene	93.9	51.7	339	92.5	19.3	—	—	—	36
Acenaphthene	71.6	24	557	68.7	78	—	—	—	40
Acenaphthylene	0.83	1.35	6.11	1.21	<0.05	—	—	—	—
Anthracene	3.8	2.12	12	0.85	<0.05	—	—	—	5
Benzo(a)anthracene	0.53	0.34	3.4	<0.05	<0.05	—	—	—	1.75
Benzo(a)pyrene	0.28	<0.05	1.27	<0.05	<0.05	—	—	—	0.7
Benzo(b)fluoranthene	0.38	<0.05	2.38	<0.05	<0.05	—	—	—	0.034
Benzo(k)fluoranthene	0.2	<0.05	0.84	<0.05	<0.05	—	—	—	0.05
Benzo(g,h,i)perylene	<0.05	<0.05	0.55	<0.05	<0.05	—	—	—	0.05
Carbazole	243	55.1	245	119	<1.00	—	—	—	—
Chrysene	0.42	0.24	2.79	<0.05	<0.05	—	—	—	0.2
Di (2-ethylhexyl) phthalate	<1.00	<1.00	<1.00	<1.00	<1.00	—	—	—	8
Dibenzo(a,h)anthracene	<0.20	<0.20	0.103	<0.50	<0.01	—	—	—	0.18
Dibenzofuran	83.6	84	202	45.1	<1.00	—	—	—	7.9
Phenanthrene	29.9	28.6	85.7	4.46	0.73	—	—	—	—
Fluoranthene	2.4	2.92	7.44	<0.50	0.2	—	—	—	1
Fluorene	56.4	32	223	31.9	0.5	—	—	—	22
Indene(1,2,3,cd) pyrene	<0.05	<0.05	0.86	<0.05	<0.05	—	—	—	0.17
Naphthalene	8800	5030	1960	1970	1620	—	—	—	140
Pentachlorophenol	<0.00001	0.00486	<0.00001	<0.00005	<0.00001	—	—	—	9
Pyrene	3.18	3.21	18.4	<0.05	<0.50	—	—	—	—

Chemical analysis of volatile organic compounds (VOCs) and semi-volatile organic compounds (SVOCs) in the eight aquifer wells.

HC (high contamination), LC (low contamination) e NC (non contamination).

The symbols - indicate absence of the contaminant.

^*^Brasilian national council of the environment.

### Creosote compound level influences the richness and diversity of aquifer wells’ bacterial community

We analyzed the influence of creosote compounds on the aquifer bacterial community by metataxonomic analyses and obtained 1,289,429 high-quality 16S rRNA sequence reads (score Phred ≥ 20 and 400 pb) from the eight aquifer wells. After removal of singletons and chimeras, 1,035,535 reads were retained, with an average of 129,441 reads per well. The rarefaction curves representing OTUs with at least 97% sequence similarity in all wells tended to an asymptote reaching 0.99 of good coverage values (Supplementary Fig. S1), indicating that the depth of sequencing was sufficient to fully cover the bacterial diversity. The result also shows that the number of different OTUs was generally higher in the non-contaminated wells NC1 and NC2, than in those with higher contamination levels.

In general, the bacterial richness in most of the contaminated wells was lower than in non-contaminated ones, according to observed OTUs numbers and Chao 1 estimator. Shannon’s diversity index considers the rare and abundant species with equal weight and Simpson’s measures the probability that two individuals, randomly selected in the sample, belong to the same species. Thus, according to both indices, the bacterial diversity was also higher in all non-contaminated wells when compared to contaminated wells (Table 2). For a better understanding of the relationship between the chemical and ecological parameters, a correlation analysis was performed. It was not possible to observe a significant correlation between bacterial richness and contaminant concentration. However, a significant and negative correlation (p-value < 0.5) was observed between the Shannon index and concentration of PAHs with 3, 4, 5 and 6 rings and between the Simpson index and PAHs with 2, 3, 4, 5 and 6 rings (Supplementary Table S1).

**Table 2:** Reads number obtained for sequencing, observed numbers of Operational Taxonomic Units (OTUs), Chao 1 richness estimator and Shannon and Simpson diversity indices for the eight aquifer wells.

Sample	N° Reads	N° OTUs	Richness estimator	Diversity index	
			Chao1	Shannon	Simpson
NC1	148,999	820	863	7.5	0.99
NC2	267,500	852	927	6.2	0.96
NC3	53,797	396	445	6.7	0.97
LC1	92,707	497	586	5.8	0.96
LC2	113,377	512	580	4.7	0.91
HC1	264,690	518	622	5.2	0.91
HC2	27,416	309	368	5.3	0.94
HC3	67,049	328	344	5.0	0.90

### Creosote level impact the aquifer wells bacterial community

**Figure 2:**
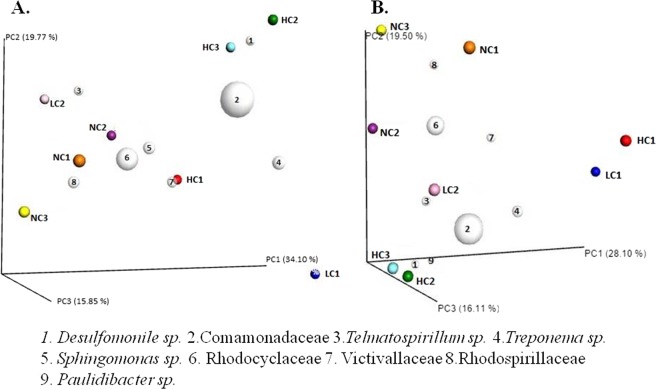
Principal Cordinate analysis (PCoA) of the bacterial communities from the eight aquifer wells. (**A**) Clustering similarity using weighted UniFrac; and (**B**) Unweighted UniFrac. The wells were represented by point symbols with different colours. The numbered white circles correspond to Operational Taxonomic Units (OTUs) that most influenced the pattern of grouping.

β-diversity analysis was carried out using the weighted and unweighted UniFrac distances, visualized in principal coordinates analysis (PCoA) biplots (Fig. 2A,B). With both metrics, close clustering was observed among the bacterial communities of most wells with some PAH contamination level and also among those without PAHs, highlighting the clear separation of the highly contaminated samples, HC2 and HC3, to the rest (Fig. 2A,B). In unweighted UniFrac, the formation of a cluster specifically of wells without contamination (NC1, NC2 and NC3) was also observed. All taxons represented by white circles were the ones that most influenced the grouping pattern of the wells, especially the *Comamonadaceae* and *Rhodocyclaceae* families, represented by larger white circles beyond the *Rhodospirilaceae* and *Victivallaceae* family and *Desulfomonile*, *Telmatospirillum*, *Treponema*, *Sphingomonas* and *Paulidicater* genus.

A total of 38 phyla, 100 classes, 161 orders, 242 families and 330 genera were found in the aquifer samples. In general, abundance of the bacterial community taxa varied according to the well sampled and PAH level. *Proteobacteria* was the most abundant phylum in all samples, with relative abundance between 36.2 and 69.8% (Supplementary Fig. 2A). *Alphaproteobacteria* and *Betaproteobacteria* were the most frequent classes in practically all samples, with relative abundance between 7.1 and 28.2% and 5.8 and 38.5%, respectively (Supplementary Fig. 2B). *Planctomycetia* was most abundant in non-contaminated wells, with relative abundance means values corresponding to 7.5% in contrast to 0.8% in contaminated wells (Supplementary Fig. 2B). *Sphirochaetes* and *Bacteroidea* were detected only in contaminated wells and *Verrucomicrobiae* was enriched specifically in the HC2 and HC3 wells with relative abundance between of 3–6% in contrast to values between 0.1–0.2% in the other wells (Supplementary Fig. 2B). The families *Rhodospirillaceae* and *Rhodocyclaceae* in general presented a high frequency in almost all wells, with relative abundance between 2.7 and 15.7% and 3.0 and 33.1% respectively, but these values are even higher in contaminated samples. Sphingomonadaceae family was most frequent in non-contaminated wells with relative abundance mean values corresponding to 12.5% in contrast to mean values of 3.0% in contaminated wells (Supplementary Fig. 2C). The high-contaminated wells (HC) showed the enrichment of the families *Geobacteriaceae*, *Porphymonadaceae*, *Verrumicrobiaceae*, *Syntrophaceae*, *Spirochaetaceae* and mainly *Comamonadaceae*, corresponding to an average of 25% of the reads (Supplementary Fig. 2C).

**Figure 3:**
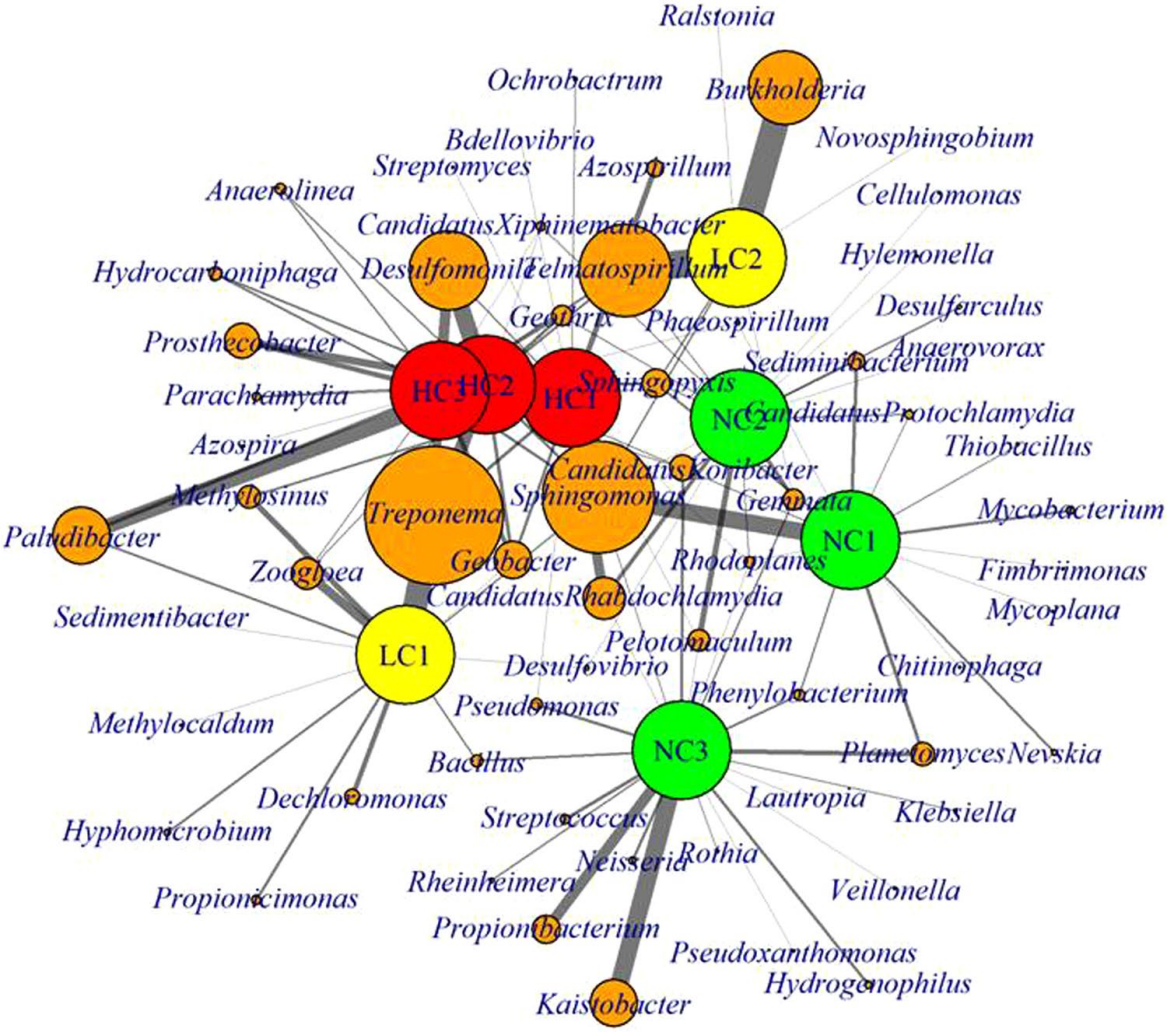
Network comparing bacterial dominant genus (>0.5%) detected in the eight aquifer wells. The non contaminated wells (NC) are represented by large the green nodes; the low contaminated wells (LC) by the large yellow nodes; the high contaminated wells (HC) by the large red nodes and the taxa by the small yellow nodes. The size of taxa nodes is proportional to the cumulative relative abundance of each taxon in all investigated samples. On the contrary, the width of line connecting wells and bacteria genus is proportional to the amount of this genus in each specific well.

The profile of genera (with relative abundance above 0.5%) was very distinct among the wells. Only the genera *Sphingomonas* was shared by all wells and several genera were shared or enriched only by wells with the same level of contamination (Fig. 3). *Phenylobacterium* and *Planctomyces* were shared only by the non-contaminated wells and this group also presented the most different genera profiles (Fig. 3). The genera *Geobacter* (*Geobacteriaceae)*, *Treponema* (*Spirochaetaceae)* and *Paludibacter* (*Porphymonadaceae)* were shared only by the wells with some contamination level and the *Desulfomoniles* genus (*Syntrophaceae)* was exclusively found in the three wells with high contamination levels (Fig. 3). Wells HC2 and HC3 presented similar genus profiles, sharing several genera exclusive of them, such as *Anaerolinea* (*Anaerolineaceae)*, *Hydrocarboniphaga* (*Sinobacteraceae)*, *Parachlamydia* (*Parachlamydaceae)* and *Prosthecobacter* (*Verrumicrobiaceae)* (Fig. 3).

**Figure 4:**
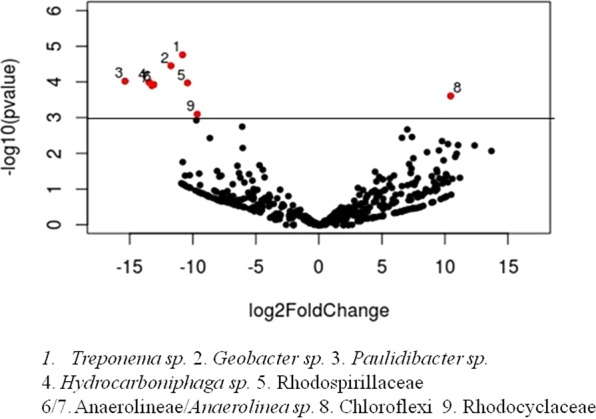
Significance analysis of the absolute abundance of the taxa for bacterial communities between high contaminated (HC) and non-contaminated (NC) wells using the edgeR analysis. In the volcano plot the taxa that differ between these groups of wells are shown above the dashed line of the y-axis, corresponding to 0.05 of p-value and the x-axis represents the fold changes in absolute abundance (log2) of the taxa.

The main alterations between the bacterial community of the wells with and without contaminants were further explored by significance analysis of the absolute abundance of taxa using edgeR package in R software (p < 0.05) and volcano plot that show a general perspective of the OTUs’ fold changes between these groups of samples (Fig. 4). This analysis confirmed the significative increase of families *Rhodospirillaceae* and *Rhodocyclaceae*, genus *Anaerolinea* of the *Anaerolineaceae* family, beyond the genera *Treponema*, *Geobacter*, *Paulidibacter* and *Hydrocarboniphaga*, on the high contamination wells (Fig. 4). In the non-contaminated wells, there was a significative increase of one non-identified genus of the *Chloroflexi* phylum.

### Different groups of creosote-PAHs directly affect the aquifer wells bacterial community

**Figure 5:**
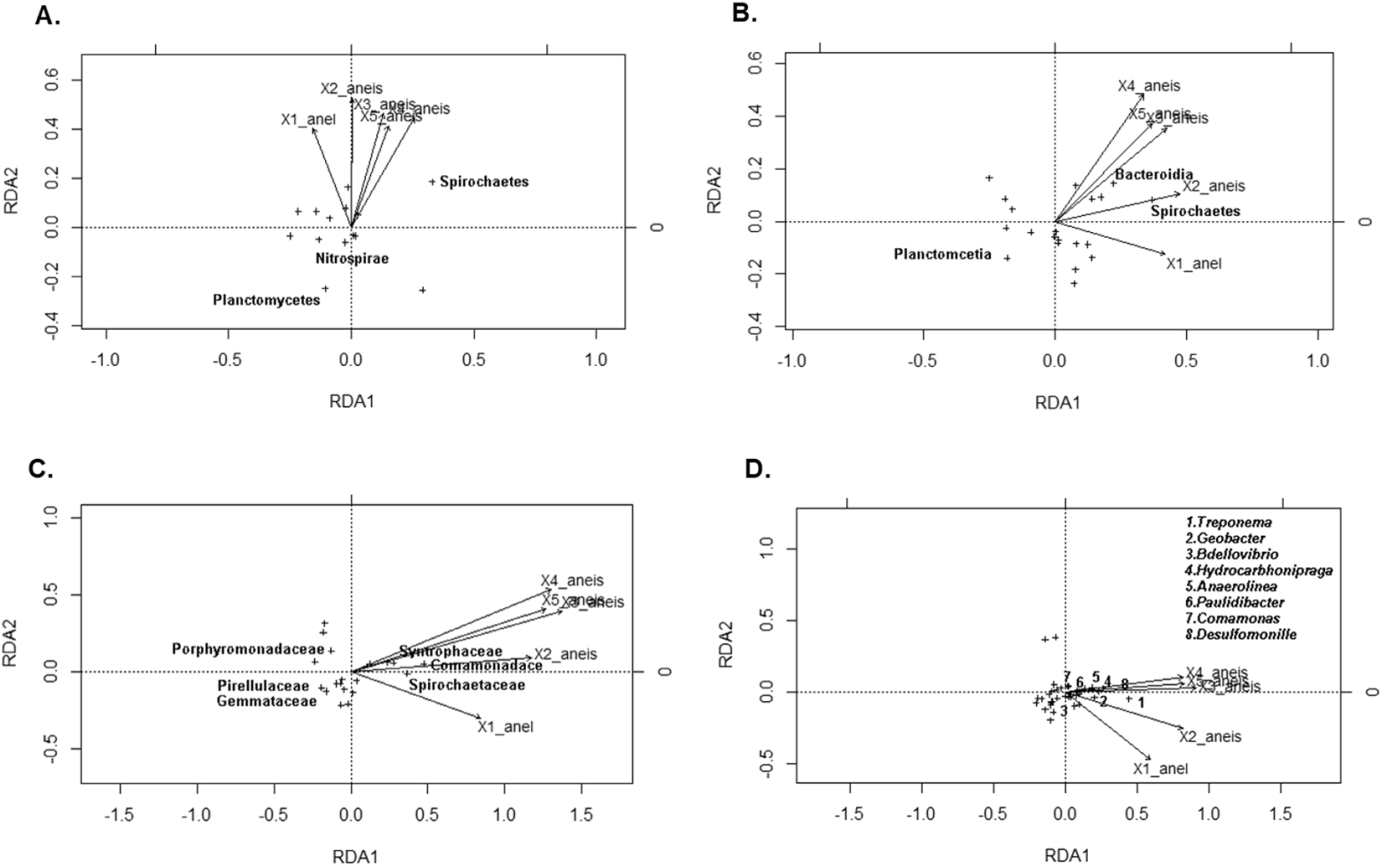
Redundancy analysis (RDA) at: (**A**) Phylum; (**B**) Class; (**C**) Family; and (**D**) Genus level. Taxa are represented by crosses and only those taxa with significant correlations (p < 0.05) with the metadata are represented in the graphs.

We used redundancy analysis (RDA) to evaluate whether the PAH levels could be contributing with structural variations on the bacterial communities. Therefore, only the taxa with significant correlations (p < 0.05) with the metadata were represented in the graphics (Fig. 5). *Planctomycetes* and *Nitrospirae* phyla were negatively affected and their abundances decreased with the increase of the PAH concentration (Fig. 5A). On the other hand, *Spirochaetes* responded positively to the increase in the contaminants concentration (Fig. 5A). The *Planctomycetia* class declined with increasing hydrocarbon concentration, whereas *Spirochaetes* and *Bacteroidia* were enriched; the first in response to an increase of PAHs with a lower number of rings (n ≤ 3) and the second in response to an increase of PAHs with a higher number of rings (n ≥ 5) (Fig. 5B). *Gemmataceae* and *Pirellulaceae* families declined with the increase in the hydrocarbon concentration but Porphyromonadaceae were correlated with an increase of PAHs with up to two rings, *Spirochataceae* and *Syntrophaceae* were associated with PAHs with up to five rings, and *Comamonadaceae* responded to the increase of all hydrocarbons, including PAHs with 6 rings (Fig. 5C). In the genus level, the enrichment of some specific groups is observed in a more pronounced way as a result of the increased contaminants concentration. The *Anaerolinea* genus was enriched with the increase of hydrocarbons with a lower number of rings, (n < 3) *Bdellovibrio*, *Comamonas*, *Desulfomonille* and *Treponema* with the hydrocarbons of intermediate size (n between 3 and 4) and *Hydrocarboniphraga*, *Geobacter* and *Paulidibacter* with all contaminants (Fig. 5D).

### Prediction of functions related to the aromatic hydrocarbons degradation from the aquifer wells bacteria metagenome

**Figure 6:**
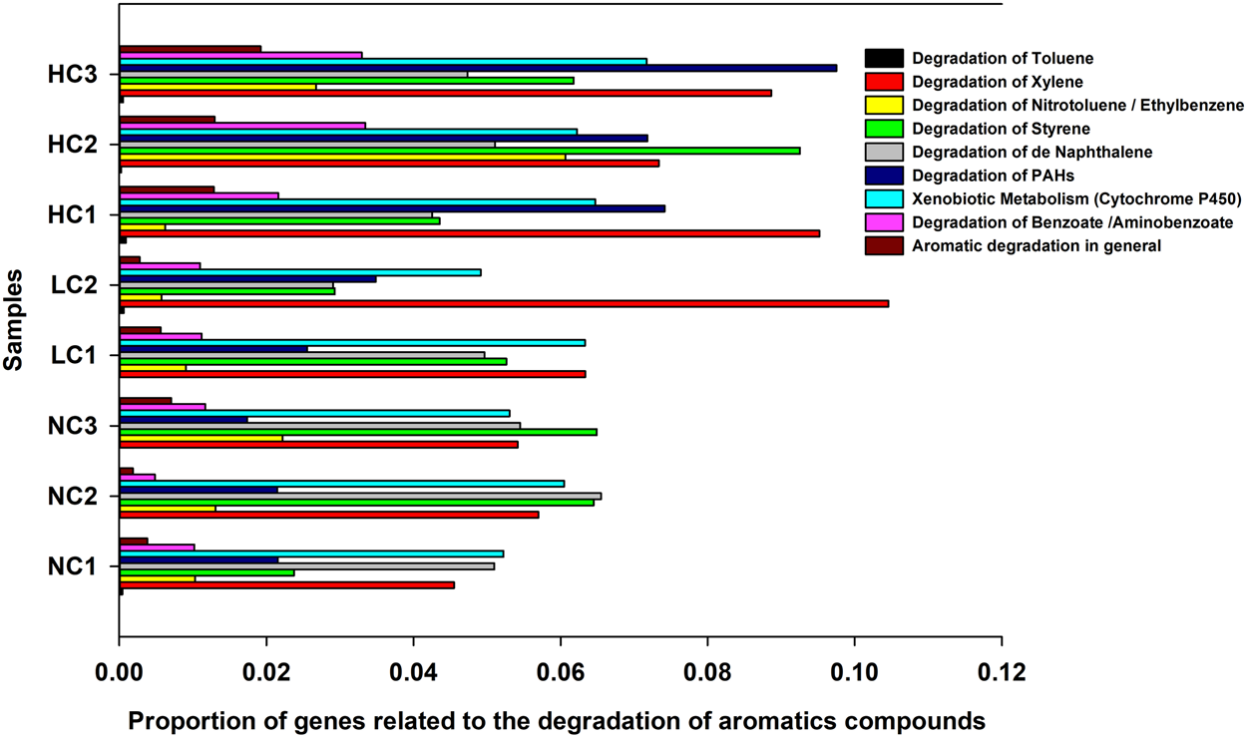
Proportion of genes related to the degradation of aromatic compounds predicted in the wells.

Most of the pathways present in the Kyoto Encyclopedia of Genes and Genomes (KEGG)^40^ third level of hierarchy were predicted in the metagenome (292 of the 328 described). In general, we did not observe large changes in the proportion of the main functions between the wells. Pathways related to the metabolism were predominant, representing more than 50% of the predicted pathways (Supplementary Fig. 3A). Within this category, the xenobiotic degradation pathways represented on average 6.6% in wells NC1, NC2 and NC3, 7.8% in wells LC1 and LC2 and more than 8.4% in wells HC1, HC2 and HC3 (Supplementary Fig. 3B). At the enzymatic level, the presence of more than 3000 enzymes was inferred, of which 234 were related to the degradation of xenobiotic compounds. Of this total, more than 100 were related specifically to the aromatics degradation, including enzymes involved in different steps of the BTEXs degradation pathways, benzoate, styrene, naphthalene and other PAHs, like the steps of catechol and protocatechuate cleavage and metabolism of these xenobiotics by Cytochrome P450. In general, there was a mean enrichment of genes encoding enzymes that participate in different stages of the degradation pathways from these aromatic compounds and their intermediates in the wells with some contamination level when compared to those that were non-contaminated (Fig. 6). Some these enriched genes were selected, highlighting the genes that participate in the synthesis of the protocatecoate, decarboxylation of phthalate, hydroxylation of salicylic acid and others (Supplementary Table 2). The Nearest Sequenced Taxon Index (NSTI), which measures the accuracy of metagenome predictions, are 0.2, 0.13, 0.16, 0.19, 0.19, 0.15, 0.12 and 0.12, respectively for the samples NC1, NC2, NC3, LC1, LC2, HC1, HC2 and HC3, so the average NSTI for the 8 samples was 0.1575.

## Discussion

The aquifer samples were analyzed regarding the concentration of 31 different VOCs and SVOCs. Although the three groups of wells presented varying profiles in relation to the distribution of these contaminants (Table 1, Fig. 1), the concentrations of many of them surpassed the research value set by Brazilian National Council for the Environment (CONAMA) Resolution 420 in most of the contaminated wells (Table 1). Among them, naphthalene and benzene represent the most worrisome contaminants; naphthalene is one of the 16 PAHs considered a priority for remediation according to the US-EPA agency because of its toxic, genotoxic, immunotoxic and carcinogenic properties and also because it is a more soluble and easily disseminated compound than PAHs with more rings^41–43^. Benzene, a monoaromatic hydrocarbon, is even more soluble than naphthalene and is the most toxic of the BTEXs, which can cause leukopenia, cancer, dizziness, tremors, and affect the central nervous system in chronic exposures^44–47^. These most soluble hydrocarbons are the common contaminants in a large number of aquifers and consequently the main problem associated with underground contamination^45–47^. They tend to bioaccumulate in different compartments of the ecosystem and their low adsorption to soil matrices contribute to their mobilization in contaminated sites^48^.

A metataxonomic approach was used in this work to characterize the structure of the microbial communities in these creosote-contaminated and non-contaminated wells. With some exceptions, according to the Chao1, Shannon and Simpson indices, which consider OTU abundance, there was a significant reduction of richness and diversity in the contaminated wells in relation to the non-contaminated ones (Table 2). So, these contaminated wells present a lower equitability of the taxa and a higher OTUs dominance. This suggests that the contaminants affected the communities structures and promoted the selection of some taxa in the samples, which is generally attributed to the differential proliferation of a few degrading microbial groups in these environments, where the contaminant becomes a large fraction of the organic carbon available to these communities^49,50^. In fact, it was observed enrichment of these specialized taxa forming a pattern that also has been observed in other studies from environmental samples with high concentrations of PAHs^51–53^. Additionally, for both diversity indices, the strongest negative correlation occurred with the group of hydrocarbons with six rings, which are more complex, recalcitrant and less susceptible to microbial degradation when compared to those with fewer rings^54^.

There were changes in the bacterial community taxa profiles of the contaminated wells in relation to the non-contaminated ones (Fig. 2). These groups of wells were separated by the β-diversity analysis using the weighted Unifrac metric, which considers the phylogenetic affiliations and relative abundance of OTUs (Fig. 2A) and unweighted UniFrac, which is sensitive to the presence and absence of taxa but not to the relative abundance of the OTUs. With this last metric, we observed the formation of a tight cluster of non-contaminated wells – NC1, NC2 and NC3 (Fig. 2B). A tight clustering is even more evident among the HC2 and HC3 wells in relation to the others, within both metrics, highlighting the similar richness and pattern of taxa between them and different from the others. This corroborates the smaller diversities calculated for these highly contaminated samples (Table 2) due to the selection of taxa caused by contaminants.

We observed that not all contaminated wells clustered together with both metrics, and even in the contaminated samples there was no formation of two groups thoroughly correlated with the two levels of contamination (Fig. 2). This confirms that the general levels of contaminants and even the contaminants predominant specifically in each well affected their bacterial communities structures. (Table 1). Although some key enzymes participate in degradation steps of different PAHs, such as the naphthalene dioxygenases (NDO)^55^, in general, the catabolic enzymes are specific to the substrate^56^. Thus, it is expected that the presence of specific hydrocarbon patterns in contaminated wells can be associated with the changes in the bacterial community structure of these wells as a whole^57^.

Although the composition of the bacterial communities varied according to the wells, in all of them, *Proteobacteria* phylum was predominant (Supplementary Fig. 2A). This phylum contains more than 400 genera, comprising approximately 30% of the species described in the Bacteria Domain and has been frequently prevalent in most studies with environmental samples, including aquifers^58–60^. Families commonly found in aquatic environments also were observed; as example the *Rhodospirillaceae*^61^ and *Rhodocyclaceae*^62^ that predominated in all wells; *Sphingomonadaceae*^63^, which was enriched in the non-contaminated wells, and *Geobacteriaceae*^64^, *Porphymonadaceae*^65^, *Verrumicrobiaceae*^66^, *Syntrophaceae*^67^, *Spirochaetaceae*^68^ and *Comamonadaceae*^69,70^, which were enriched in the contaminated wells (Supplementary Fig. 2C).

In general, we observed that the non-contaminated wells presented more diverse profiles of dominant genera and, consequently, a smaller number of genera were shared among them when compared to the high-contaminated wells, whose profiles of dominant genera were more similar (Fig. 3). As already mentioned, environments with selective pressure caused by the presence of extremely toxic contaminants, such as PAHs, tend to be less diverse. Therefore, it is common that a smaller number of genera could be able to survive under these conditions, and consequently that contaminated samples share these enriched taxa. This pattern is also reported in other studies with hydrocarbon-contaminated and non-contaminated samples^71,72^. In relation to the enriched genera *Geobacter*, *Treponema*, *Paludibacter*, *Desulfomoniles*, *Anaerolinea, Hydrocarboniphaga*, *Parachlamydia* and *Prosthecobacter*, some of them were the ones that most contributed to the grouping of wells in β-diversity analysis (Fig. 2) and were higher (p < 0.05) in the group of high-contaminated wells, according to the edgeR analysis as showing in the volcano plot (Fig. 4).

The influence of the presence and concentration of the contaminants on the previously mentioned enriched taxa, mainly at genera level, are even more evident through the RDA analysis, in which practically all of them correlated positively and significantly with the different groups of contaminants (Fig. 5). All these genera have members already characterized, directly or indirectly, regarding the degradation of xenobiotics by aerobic or anaerobic pathways. Strains of *Comamonas* able to degrade phenol^73^ and 3-chloroaniline^74^ were isolated from activated sludge and able to degrade naphthalene, phenanthrene and anthracene were isolated from river sediment^75^. *Geobacter* isolates able to oxidize aromatic compounds have already been described in contaminated aquifers^76,77^. The genus was associated with the anaerobic degradation of monoaromatic hydrocarbons, such as toluene, phenol, p-cresol and o-xylene^78^. *Bdellovibrio* isolates were enriched in a BTEX-fed reactor for water treatment^79^ and *Hydrocarboniphaga* isolates in petroleum-contaminated soils^80,81^. *Desulfomonille* is the only known anaerobic genus capable of promoting the degradation of chlorinated aromatic compounds, such as benzoates in pure culture^82,83^. Bacteria of the genus *Anaerolinea* are strictly anaerobic microorganisms present in high frequency in aquifers, sediments and other environments contaminated with hydrocarbons, suggesting that members of this genus play a role in the anaerobic biodegradation of these contaminants, although this role is unclear^84–86^. The genera *Treponema* and *Paludibacter*, which showed a positive correlation with the presence of PAHs, have not yet been directly characterized as to the ability of hydrocarbon degradation. But interestingly, in more than one work, they are reported as genera that tend to be successors of microorganisms directly involved in the degradation of hydrocarbons after the reduction of the contaminant in the environment^87,88^.

As one of the main strategies to achieve the rapid and satisfactory degradation of PAHs in a bioremediation project is to precisely stimulate the growth of degrading bacteria present in the site, the occurrence of these degrading groups in high frequency opens promising prospects for this process feasibility. After metagenome gene prediction of the wells using the PICRUSt program, a greater proportion of xenobiotic degradation-related pathways (Supplementary Fig. 3B) and many enzyme-encoding genes involved in aromatics and their intermediates degradation’s pathways was observed in the highly contaminated wells (Fig. 6, Supplementary Table 2). This is due to the community structure of the dominant bacteria in these wells, which are enriched in taxa known to potentially degrade PAHs. In the *Comamonas* genus, as an example, the presence of alpha subunit genes for PAH dioxygenases enzymes was already detected^89^ and Zylstra *et al*.^90^ showed that the genus has a little distinctive initial PAH dioxygenase, compared to the highly conserved *Pseudomonas* naphthalene dioxygenase genes (nahAaAbAcAd). For a *Geobacter metallireducens* isolate, the production of benzylsuccinate synthase (BSS) enzyme was reported, which catalyzes the addition of the methyl carbon of toluene to the double bond of fumarate^91^. This is a stage in anaerobic toluene degradation and involve in the anaerobic activation of a variety of other compounds, including m-xylene and 2-methylnaphthalene^91^. Some alkB genes, encoding alkane 1-monooxygenase, a key enzyme responsible for the initial oxidation of inactivated alkanes, are commonly found in bacteria of the genera *Bdellovibrio*^92^. The *Desulfomonille tiedjei* species have genes that encode enzymes capable of promoting the reductive dehalogenation of chlorinated aromatic compounds^83^. The accuracy of metagenome predictions depends on how closely related the microbes in a given sample are to microbes with sequenced genome representatives, with lower NSTI values indicating a closer mean relationship. The NSTI observed for the samples ranged between 0.12 and 0.2. For comparison, Langille *et al*.^39^ found lowest NSTI values in human-associated samples (0.03 ± 0.2), but diverse communities such as soil still produced reliable results with a much higher NSTI value (0.17 ± 0.02). Additionally, the NSTI values obtained in this work were similar and in agreement with that found in previous studies with environmental samples^93–96^. Thus, it is suggested that the diverse and complex communities in the aquifer samples provided a satisfactory data set to examine predictions from PICRUSt. This prediction approach does not replace the metatranscriptomic profiling of the samples. However, it has been shown to be a good tool to indicate the functional potential of bacterial communities under different conditions^97,98^.

The results show that the bacterial community of the area is very diverse and dynamic and that the presence of the contaminants was able to profoundly alter its structure, with the enrichment of several groups of bacteria in the wells contaminated with creosote in relation to the non-contaminated ones. Most of these groups of bacteria have been directly or indirectly related, in several studies, to the survival capacity and degradation of different PAHs that compose this contaminant both through aerobic and anaerobic degradation pathways. Thus, it is possible to confirm the presence of microorganisms with degradation potential in the study area, which can be biostimulated *in situ* to accelerate the degradation of these contaminants under appropriate nutritional and physic-chemical conditions.

## Material and Methods

### Experimental design and aquifer wells collection

Samples of the aquifer liquid fraction were collected in a creosote-contaminated area located in the city of João Neiva, in July 2015 in the state of Espírito Santo under the coordinates 19°44′03″S and 40°21′50″W (Supplementary Fig. S4). In this area, there was an old railway sleeper’s treatment and maintenance station (TMS) that used creosote as preservative for woods. Samples were collected at locations H1, H2, H3, H4, H5, H6, H7 and H8 (Supplementary Fig. S4) using disposable bailer-type samplers. The wells where the samples were collected were drilled between 2009 and 2011 with depths varying between 4 and 20 m and were designed for sampling groundwater, nucleic acid extraction and chemical analyses. Prior to sampling, superficial water corresponding to at least three bailer volumes was collected and discarded, and the collection flasks were acclimatized three times with the water from the wells. Subsequently, 5L of collected groundwater were prefiltered on-site using a vacuum filtration pump connected to a stainless steel filter device (Millipore, Watford, United Kingdom) equipped with polycarbonate membranes with pores of 20 μm in diameter (47 mm diameter; Millipore) for removal of particles and dirt. Then, the samples were subjected to two successive filtrations using polycarbonate membranes with pores of 1.2 and 0.22 μm in diameter (47 mm diameter; Millipore). Filters were immediately transferred to dry ice and stored at −80 °C until nucleic acid extraction. The criterion for choosing the wells was the mean concentration of 31 different VOCs and SVOCs compounds (Table 1).

### Chemical analysis of the aquifer wells

The VOC and SVOC concentrations in the samples were analyzed according to the US-EPA 8260B and US-EPA 8270C methodologies, respectively. The wells were grouped in relation to the concentrations of the contaminants through PCA using PAST software version 3.04^99^.The results of the analyses were compared with the list of Guidance Values for Soil and Groundwater of CONAMA resolution 420/2009, of December 28, 2009, which establishes the guidelines and procedures for the protection of soil quality and environmental management of chemical contaminated areas.

### Metagenomic DNA extraction

DNA was extracted from the filter membranes using the PowerWater DNA Isolation Kit (MoBio Laboratories, Carlsbad, CA) according to the manufacturer’s instructions. The DNA quality was evaluated in 1% agarose gel electrophoresis stained with ethidium bromide and the quantification was performed by reading the absorbance at 260 nm in a spectrophotometer (NanoDrop Technologies, Wilmington, DE).

### Polymerase chain reaction and sequencing

The PCR reaction was performed to amplify the V3 and V4 regions of the 16S subunit of the rRNA gene for bacteria, using the primers oligonucleotides 341F and 806R^100^. In addition, the forward-5′TCGGGGGCA GCG TCA GAT GTG TAT AAG AGA CAG 3′ and reverse-5′GTC TCG TGG GCT CGG AGA TGT GTA TAA GAG ACA G3′ ‘overhang’ sequences compatible with the indexes and adapters of the Illumina Miseq platform were added to primers oligonucleotides using the conditions described in the protocol proposed by Illumina. Initially the PCR reaction was performed in a final volume of 25 μL containing 12.5 μL of 2× KAPA HiFi HotStart ReadyMix, 5 μL of each primer (1 pmol μL-1) and 2.5 μL of the DNA (15 ng μL-1). Amplification was performed with initial denaturation at 95 °C for 3 min, followed by 30 cycles at 95 °C for 30 sec, annealing at 60 °C for 30 sec and extension at 72 °C for 30 sec. The final extension was conducted at 72 °C for 5 min. Amplicons were purified with AMPure XP DNA purification beads (Beckman Coulter Genomics, Danvers, MA, United States) according to the manufacturer’s instructions. Subsequently, the amplicons were ligated to a specific index pair (N7 and S5) for each sample during a second PCR: to a final volume of 25 μL, 12.5 μl of 2× KAPA HiFi HotStart ReadyMix, 3 μl of each Nextera XT index, 2.5 μL of the purified product from the first PCR and 4 μL of ultrapure water. Amplification was performed with initial denaturation at 95 °C for 3 min, followed by 8 cycles at 95 °C for 30 sec, annealing at 55 °C for 30 sec, extension at 72 °C for 30 sec. and final extension at 72 °C for 5 min. The product of the second PCR was purified with AMPure XP DNA purification beads (Beckman Coulter, Danvers, MA, USA), following the manufacturer’s instructions. After purification, the amplicons were evaluated for the quality and size of the bands using Bioanalyzer DNA 1000 chip (Agilent, Santa Clara, CA, United States). Then, the samples were quantified by qPCR using KK4824-Kappa Biosystems kit (Biosciences, Woburn, MA, United States) in the Step one real time PCR (Applied Byossystems) following the manufacturer’s instructions. The DNA concentration expressed in nM was calculated based also on the DNA amplicons size according to the following equation: (concentration in ng/µl)/(660 g/mol × average library size) × 10^6^. The resulting libraries were diluted using Resuspension Buffer (RSB) to a concentration of 4 nM. Then, 5 µl aliquots from each library were mixed to form pooled libraries, which were denatured with 0,2 N NaOH, diluted with HTI hybridization buffer, and then heat denatured before the sequencing on the Illumina MiSeq platform using the V3 kit, with 2 × 300 bp paired-end. The run also included 5% PhiX to serve as an internal control.

### Sequence analysis

The sequences obtained were demultiplexed to group the samples according to the index set. The quality of the sequences was then evaluated based on the MiSeq Illumina sequencer quality filter, excluding sequences with low-quality values. After this initial screening, the sequences were converted to the FASTq format. The following analyses were based on the pipeline developed by the Brazillian microbiome project^101^, with modifications. Initially, sequences in the FASTq format were trimmed for quality, using the Phred parameter (score ≥ 15 into a sliding window composed of 4 bp) using Trimmomatic v0.32^102^ and truncated to a size of 400 bp. Then, the singletons were removed, and the chimeric sequences filtered using the USEARCH^103^. Taxonomic classification of the reads was based on the Greengenes database for a minimum similarity of 97% using the QIIME package^104^. Raw data from 16S rRNA amplicon Illumina sequencing were submitted to the GenBank databases under accession number PRJNA429484.

### Statistical analysis

The taxonomic classification, good coverage and alpha and β-diversity analysis were performed using the core_diversity pipeline of the QIIME package^104^. The rarefaction curves were constructed based on the number of OTUs observed per sample, and the sampling saturation was evaluated by good coverage analysis. The Chao1 index was calculated to determine the richness of the samples, the Shannon and Simpson indices to determine the alpha diversity, and the correlation of these parameters with the contaminant concentration was evaluated using the Spearman coefficient in the Minitab 17 program. β-diversity was performed by PcoA using the weighted and unweighted Unifrac distance matrix^105^. The igraph and qgraph package in R program were used to construct a network visualizing the profile of genera shared or not among the wells. The significance analysis of the absolute abundance of the taxa among the group with high contamination (HC samples) and without contamination (NC samples) (p < 0.05) was performed using the EdgeR package in the R program. In order to evaluate the effect of the contaminants in the distribution of the bacterial community at the phylum, class, family and genus levels, a RDA was performed using the vegan package in the R program after the data was normalized by the Hellinger transformation, which minimizes the effect of zeros on community data^106^. The potential functions in the bacterial communities of the samples were evaluated with the PICRUSt program^39^. This program uses an ancestral reconstruction algorithm to estimate the number of genes of each gene family in different bacteria, based on its available phylogeny and genomes. In the initial step, the program predicts what genes are present in organisms that have not yet been sequenced based on the genes observed in their sequenced evolutionary relatives. The next step is a metagenome inference combining the gene content predictions for all microbial taxa with the relative abundance of 16S rRNA genes in the samples, corrected for expected 16S rRNA gene copy number. The process generates the expected abundances of gene families in the entire communities of the samples^39^. The functional classification scheme used in this work was the KEGG Orthology (KOs)^107^ and three levels of information of metabolic pathways were included. The NSTI score was used as an indicator for the accuracy of PICRUSt prediction results.

## Supplementary information

Supplementary figures and tables

## Data Availability

The datasets generated during and/or analyzed during the current study are available in the to the GenBank databases under accession number PRJNA429484.
