# Supplementary material for: Metataxonomic analyses reveal differences in aquifer bacterial community as a function of creosote contamination and its potential for contaminant remediation
Source: Sci Rep. 2019 Aug 13;9:11731. doi: 10.1038/s41598-019-47921-y (PMC6692397; doi:10.1038/s41598-019-47921-y)

**Metataxonomic analyses reveal differences in aquifer bacterial community as a function of creosote contamination and its potential for contaminant remediation**

Aline Daniela Lopes Júlio^1^, Ubiana de Cássia Mourão Silva^1^, Julliane Dutra Medeiros^2^, Daniel Kumazawa Morais^3^, Vera Lúcia dos Santos^1^*

^1^Laboratory of Applied Microbiology, Department of Microbiology, Institute of Biological Science, Universidade Federal de Minas Gerais. Adress: Avenida Presidente Antônio Carlos, 6627 – Pampulha / ICB, Bloco F4, sala 159. Belo Horizonte – MG, C.P. 486, 31270-901, Brazil.

^2^Biosystems Informatics and Genomics Group, René Rachou Research Center / Fiocruz-MG, Belo Horizonte, MG-Brazil.

^3^Laboratory of Environmental Microbiology, Institute of Microbiology of the Czech Academy of Sciences – CAS, Prague, Czech Republic.

*Author correspondence E-mail: verabio[@gmail.com](mailto:domingues.vtrs@gmail.com).

**Supplementary Figure S1 online:** Bacterial (**A**) Rarefaction curves and (**B**) Good coverage values bacteria were calculated according observed Operational Taxonomic Units (OTUs) in the eight aquifer samples using QIIME software.


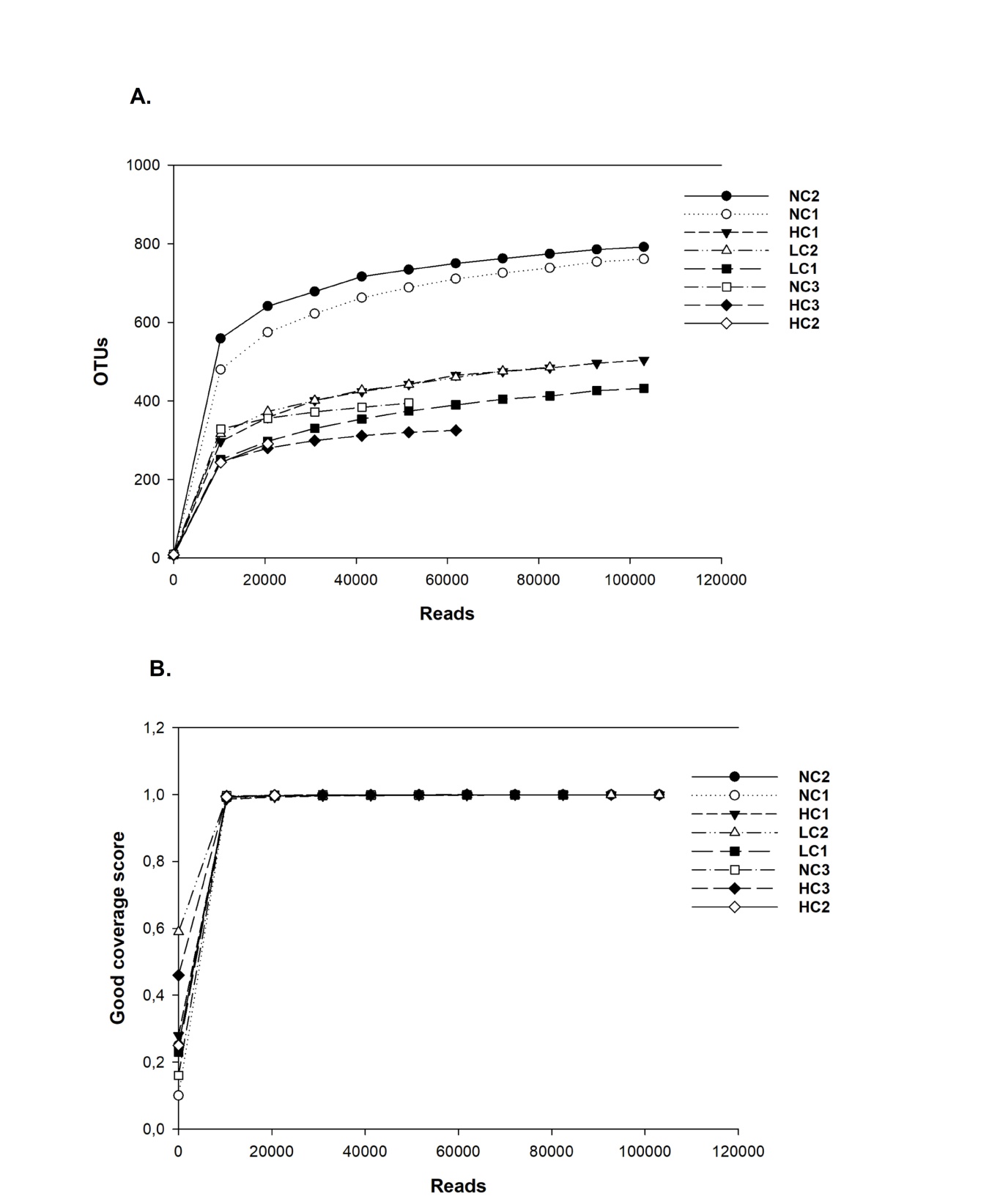


**Supplementary Table S1 online:** P-value and Spearman correlation coefficients obtained in the correlation analysis between the diversity of Operational Taxonomic Units (OTUs) calculated for the eight aquifer wells and the metadata.

| PAHs | Shannon | | Simpson | |
| --- | --- | --- | --- | --- |
|  | P-value | Coefficient | P-value | Coefficient |
| 2 rings | 0,076 | -0.659 | 0,040 | -0,728 |
| 3 rings | 0,050 | -0,708 | 0,017 | -0,803 |
| 4 rings | 0,016 | -0,805 | 0,004 | -0,877 |
| 5 rings | 0,050 | -0,708 | 0,017 | -0,803 |
| 6 rings | 0,008 | -0,848 | 0,004 | -0,878 |

**Supplementary Figure S2 online:** Relative abundance of bacterial: (**A**) Phyla; (**B**) Classes; and (**C**) Families in eight aquifer wells. Taxa occurring in at least one sample with relative abundance greater than 2% were considered.

**
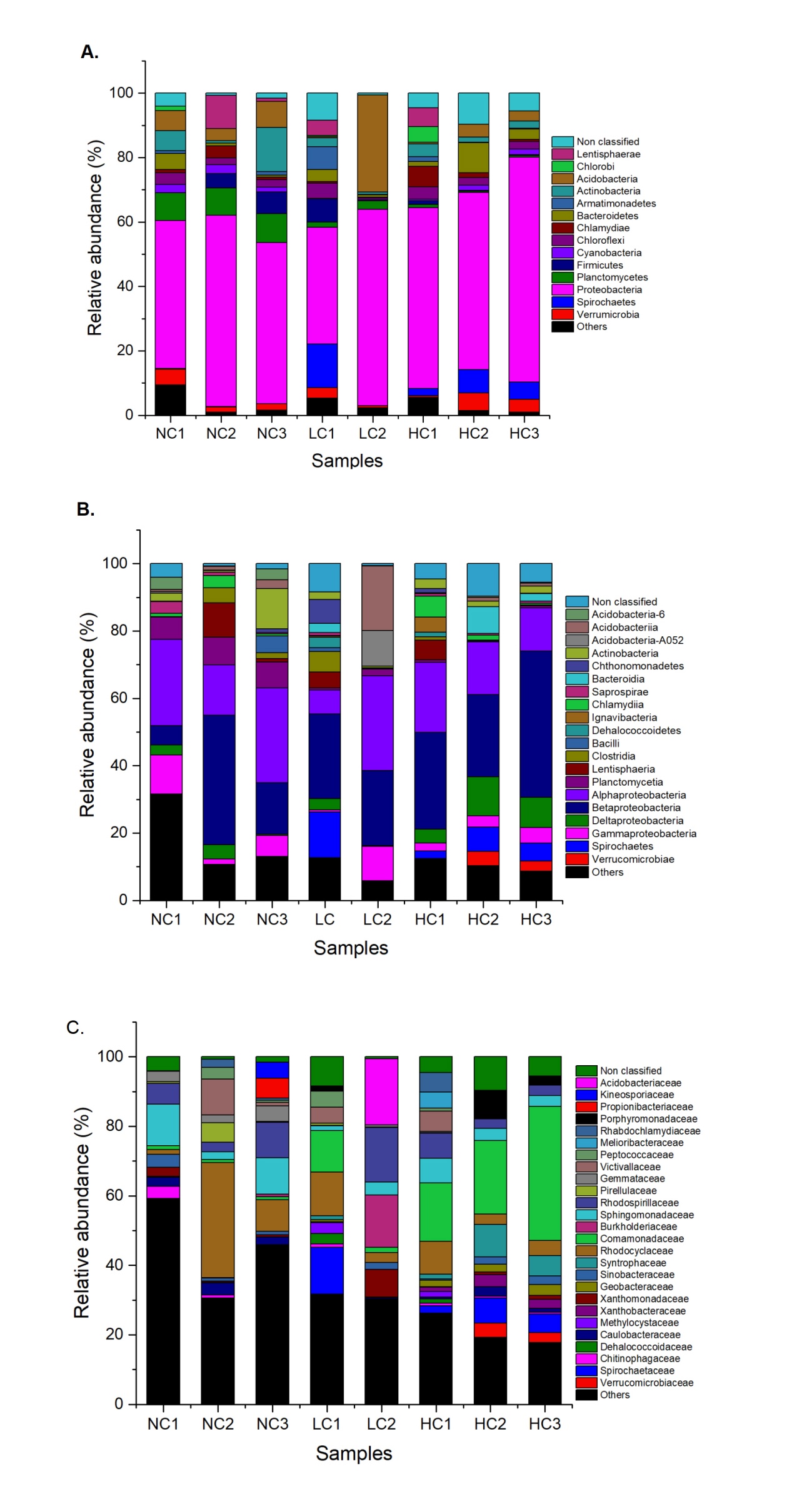
**

**Supplementary Table S2 online:** Selection and the relative number of some predicted genes involved in the degradation of aromatic compounds enriched in the metagenome of the contaminated wells.

| **Função** | **KO** | **Descrição KEGG [Nº EC]** | **NC1** | **NC2** | **NC3** | **LC1** | **LC2** | **HC1** | **HC2** | **HC3** |
| --- | --- | --- | --- | --- | --- | --- | --- | --- | --- | --- |
| Degradation of Xylene | K01821 | praC, xylH; 4-oxalocrotonate tautomerase [EC:5.3.2.6] | 0.0329 | 0.0265 | 0.0325 | 0.0516 | 0.0477 | 0.0468 | 0.0467 | 0.0550 |
|  | K01617 | dmpH, xylI, nahK; 2-oxo-3-hexenedioate decarboxylase [EC:4.1.1.77] | 0.0185 | 0.0210 | 0.0149 | 0.0112 | 0.0061 | 0.0288 | 0.0202 | 0.0260 |
| Degradação de Nitrotoluene / Ethylbenzene | K00632 | fadA, fadI; acetyl-CoA acyltransferase [EC:2.3.1.16] | 0.0091 | 0.0057 | 0.0222 | 0.0103 | 0.0131 | 0.0062 | 0.0607 | 0.0268 |
| Degradation of Styrene | K01800 | maiA, GSTZ1; maleylacetoacetate isomerase [EC:5.2.1.2] | 0.0189 | 0.0131 | 0.0166 | 0.0072 | 0.0184 | 0.0224 | 0.0207 | 0.0255 |
| Degradation of de naphthalene | K04072 | adhE; acetaldehyde dehydrogenase / alcohol dehydrogenase [EC:1.2.1.10 1.1.1.1] | 0.0121 | 0.0139 | 0.0111 | 0.0124 | 0.0076 | 0.0210 | 0.0196 | 0.0268 |
| Degradation of PAHs | K00480 | salicylate hydroxylase [EC:1.14.13.1] | 0.0103 | 0.0230 | 0.0121 | 0.0108 | 0.0147 | 0.0311 | 0.0319 | 0.0403 |
|  | K04102 | pht5; 4,5-dihydroxyphthalate decarboxylase [EC:4.1.1.55] | 0.0013 | 0.0016 | 0.0013 | 0.0031 | 0.0029 | 0.0116 | 0.0124 | 0.0171 |
|  | K04100 | ligA; protocatechuate 4,5-dioxygenase, alpha chain [EC:1.13.11.8] | 0.0069 | 0.0051 | 0.0020 | 0.0038 | 0.0020 | 0.0155 | 0.0132 | 0.0198 |
|  | K04101 | ligB; protocatechuate 4,5-dioxygenase, beta chain [EC:1.13.11.8] | 0.0071 | 0.0051 | 0.0020 | 0.0039 | 0.0020 | 0.0159 | 0.0143 | 0.0204 |
| Xenobiotic Metabolism (Cytochrome P450) | K04097 | HPGDS; prostaglandin-H2 D-isomerase / glutathione transferase [EC:5.3.99.2 2.5.1.18] | 0.0257 | 0.0236 | 0.0196 | 0.0208 | 0.0218 | 0.0332 | 0.0340 | 0.0319 |
| Degradation of Benzoate /Aminobenzoate | K03464 | catC; muconolactone D-isomerase [EC:5.3.3.4] | 0.0020 | 0.0003 | 0.0017 | 0.0009 | 0.0033 | 0.0043 | 0.0026 | 0.0022 |
|  | K01857 | pcaB; 3-carboxy-cis,cis-muconate cycloisomerase [EC:5.5.1.2] | 0.0072 | 0.0048 | 0.0099 | 0.0042 | 0.0015 | 0.0153 | 0.0150 | 0.0194 |
|  | K04109 | hcrB, hbaD; 4-hydroxybenzoyl-CoA reductase subunit beta [EC:1.3.7.9] | 0.0015 | 0.0008 | 0.0002 | 0.0015 | 0.0000 | 0.0033 | 0.0078 | 0.0055 |
|  | K07539 | oah; 6-oxocyclohex-1-ene-carbonyl-CoA hydrolase [EC:3.7.1.21] | 0.0004 | 0.0026 | 0.0000 | 0.0012 | 0.0000 | 0.0036 | 0.0080 | 0.0059 |
| Aromatic degradation in genera | K00529 | hcaD; 3-phenylpropionate/trans-cinnamate dioxygenase ferredoxin reductase component [EC:1.18.1.3] | 0.0050 | 0.0015 | 0.0034 | 0.0051 | 0.0042 | 0.0116 | 0.0239 | 0.0126 |
|  | K01826 | hpaF, hpcD; 5-carboxymethyl-2-hydroxymuconate isomerase [EC:5.3.3.10] | 0.0044 | 0.0052 | 0.0047 | 0.0093 | 0.0017 | 0.0167 | 0.0150 | 0.0199 |
|  | K05921 | hpaG; 5-oxopent-3-ene-1,2,5-tricarboxylate decarboxylase / 2-hydroxyhepta-2,4-diene-1,7-dioate isomerase [EC:4.1.1.68 5.3.3.-] | 0.0082 | 0.0107 | 0.0107 | 0.0107 | 0.0223 | 0.0172 | 0.0171 | 0.0235 |
|  | K05709 | hcaF, hcaA2; 3-phenylpropionate/trans-cinnamate dioxygenase subunit beta [EC:1.14.12.19] | 0.0100 | 0.0038 | 0.0074 | 0.0040 | 0.0021 | 0.0134 | 0.0153 | 0.0221 |
|  | K05708 | hcaE, hcaA1; 3-phenylpropionate/trans-cinnamate dioxygenase subunit alpha [EC:1.14.12.19] | 0.0056 | 0.0028 | 0.0071 | 0.0038 | 0.0018 | 0.0129 | 0.0130 | 0.0192 |

**Supplementary Figure S3 online:** **A**) Proportion of predicted genes in the metagenome of the wells categorized into groups according to their functions; and **B**) Proportion of biodegradation pathways genes within metabolism category predicted in the wells.

**
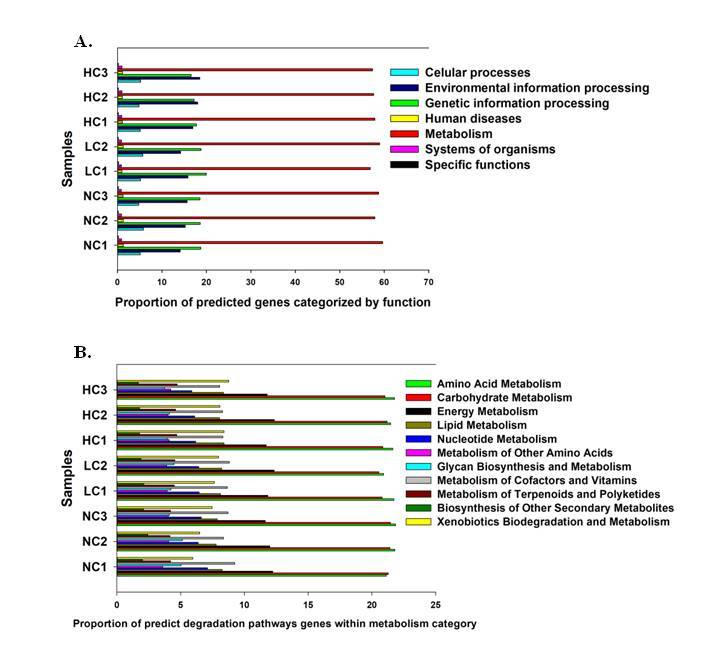
**

**Supplementary Figure S4 online:** Satellite image of the old sleepers’ treatment and maintenance station (TMS) in João Neiva/ES/Brazil and diagram of the wells sampling locations, which are circled in red.


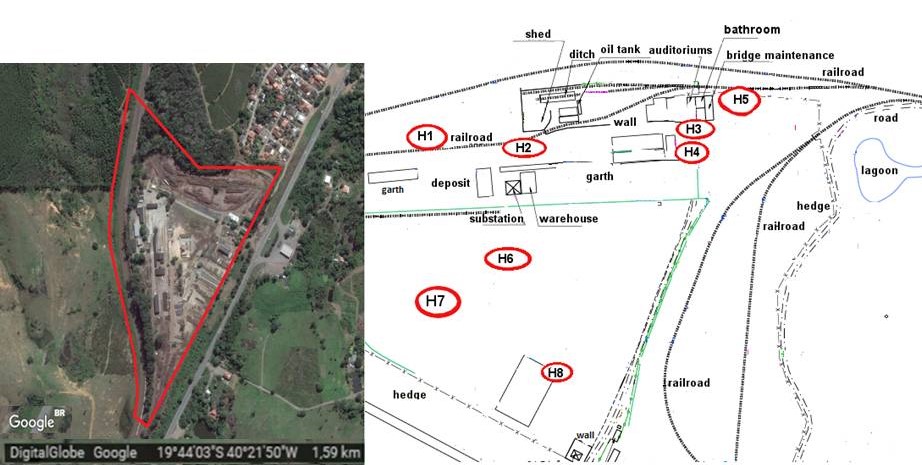

Supplement: Supplementary file 1 — Supplementary figures and tables [file 41598_2019_47921_MOESM1_ESM.docx]
